# A new image encryption algorithm based on the OF-LSTMS and chaotic sequences

**DOI:** 10.1038/s41598-021-85377-1

**Published:** 2021-03-18

**Authors:** Yi He, Ying-Qian Zhang, Xin He, Xing-Yuan Wang

**Affiliations:** 1 City Institute, Dalian University of Technology, Dalian, 116600 China; 2 School of Information Science & Technology, Xiamen University Tan Kah Kee College, Zhangzhou, 363105 China; 3 Dalian University of Technology, Dalian, 116024 China; 4 School of Information Science and Technology, Dalian Maritime University, Dalian, 116026 China

**Keywords:** Computer science, Information technology

## Abstract

In this paper, a novel image encryption algorithm based on the Once Forward Long Short Term Memory Structure (OF-LSTMS) and the Two-Dimensional Coupled Map Lattice (2DCML) fractional-order chaotic system is proposed. The original image is divided into several image blocks, each of which is input into the OF-LSTMS as a pixel sub-sequence. According to the chaotic sequences generated by the 2DCML fractional-order chaotic system, the parameters of the input gate, output gate and memory unit of the OF-LSTMS are initialized, and the pixel positions are changed at the same time of changing the pixel values, achieving the synchronization of permutation and diffusion operations, which greatly improves the efficiency of image encryption and reduces the time consumption. In addition the 2DCML fractional-order chaotic system has better chaotic ergodicity and the values of chaotic sequences are larger than the traditional chaotic system. Therefore, it is very suitable to image encryption. Many simulation results show that the proposed scheme has higher security and efficiency comparing with previous schemes.

## Introduction

With the rapid development in digital connectivity, the shared information like audio, video, image is widely spread on the network, and the security of data information is facing serious challenges. So, data security technologies like data encryption, digital signature have been extensively studied to protect data information from potential threats. Image as an important carrier of information plays a vital role in communication due to its vivid and intuitive characteristics. Therefore, the research of image encryption technology is particularly important^1–43^.

Permutation and diffusion are usually two separate stages in the image encryption^12,16–24,28^. In reference^18^ the multi-level chaotic map formed by three levels was applied to permutation and diffusion, so as to achieve good image encryption effect. Mondal et al.^19^ designed an image encryption algorithm based on pixel level scrambling in the confusion layer and bit XOR operation in the diffusion layer. At the same time, they also proposed a new two-dimensional cross chaotic map. Gopalakrishnan et al.^22^ applied the two-dimensional hyperchaotic map in the permutation stage, and the hybrid chaotic map is applied in the diffusion stage, thus the image encryption process is completed. However, due to the separation of image permutation and diffusion in traditional algorithms, the amount of data needed to be processed in the image increases significantly, which limits the efficiency of image encryption. In recent years, some researches on image encryption based on chaotic sequences and network structures have been proposed^27, 28,33,44^. Maddodi et al.^27^ generated a pseudo-random sequence generator with neural network and chaotic sequence, and then combined DNA rules for image encryption. In order to enhance the security of the financial system, Pan et al.^28^ proposed an encryption scheme which combines the hybrid chaotic system and the deep network structure to realize the dynamic image encryption technology. The above algorithms make a new attempt on image encryption, but the encryption efficiency is not high, such as the histogram effect is not ideal, the information entropy is not high, and the encryption time is long etc.

The Long Short-Term Memory (LSTM) network structure has good application effect in many fields, especially in the field of artificial intelligence such as natural language processing, image segmentation, image classification, etc. At the same time, LSTM network structure is a kind of extended structure of Recurrent Neural Network (RNN), which can effectively deal with the problem of sequence transformation. In the process of image processing, the original image is often transformed into a series of pixel sequences. Therefore, image processing can be regarded as a sequence transformation process, which is effectively processed by LSTM^29–32,45–50^. Gregor et al.^45^ proposed an image generation network based on the LSTM structure in the image generation task, which can effectively generate the image corresponding to the input image in an iterative way. In the task of brain image segmentation, Marijn et al.^46^ introduced a multi-dimensional LSTM network based on pixel by pixel pyramid operation. Zhou et al.^47^ proposed a spectral space-based LSTM network in the hyperspectral image classification task, which has good performance by inputting the local pixel block composed of core pixels determined by PCA into LSTM. Aslan et al.^48^ applied three different LSTM in the ultrasonic image analysis task to construct the tracker system, which achieved high accuracy in the test. LSTM can process images in sequence mode, and most of the LSTM used are input and output of sequence (with equal length) and synchronous. Image encryption is an important research field of image processing. In the process of image encryption, the original image can be transformed from top to bottom, from left to right, and then the pixel sequence can be input into the structure for a series of encryption algorithm operations. Therefore, the image encryption/decryption process can be regarded as a series of encoding/decoding processes. However, the image encryption algorithm requires reversibility, and the matrix multiplication of traditional LSTM structure can not meet this requirement. Therefore, this paper takes the network structure of LSTM as the basic framework, and uses XOR operation to replace the traditional LSTM calculation method. At the same time, the parameters in this algorithm are derived from chaotic sequences, which do not need to be changed. That is to say, after entering the algorithm, the encrypted image can be obtained after only one forward propagation. Therefore, the structure of the algorithm is called as the Once Forward Long Short Term Memory Structure (OF-LSTMS). The OF-LSTMS can not only change the pixel value and the pixel position information at the same time, but also retain the memory ability of the structure to the previous information. It can effectively resist malicious attacks and is more suitable for image encryption. The simulation results show that the encryption algorithm has the advantages of high information entropy, ideal histogram and low time cost, especially for large-scale images.

Chaos system has a series of characteristics such as good ergodicity, sensitivity to initial state and control parameters. These characteristics are highly compatible with the characteristics of cryptosystem. In 1989, Matthews^34^ proposed a generalized logistic map, and encrypted the text data by pseudo-random sequence generated by the map. This is the first time that chaos system is applied in the field of encryption. In 1998, Fridrich^35^ used discrete chaotic mapping to scramble the image pixels, and proposed an image encryption system based on chaos. During the last three decades, there have been significant interests in the image encryption algorithm based on chaos system^1–15,18–23,25,28,34–43,51,52^. Zhang et al.^52^ analyzed the dynamic characteristics of the fractional-order chaotic system in detail and designed an efficient image encryption algorithm. Experimental results show that the chaotic sequences generated by the system are very suitable for image encryption. Tsafack et al.^11^ generated the chaotic behavior through a circuit network, and proposed an image encryption protocol based on chaotic sequence which has a significant effect on network image encryption. El-Latif et al.^1^ applied chaotic systems to design a new cryptosystem, each of which generates periodic cycles. In addition, quantum random walks were applied in the design of cryptosystem, which solves the periodicity problem in traditional cryptosystem. Wang et al.^25^ obtained a new spatiotemporal chaos system by applying three different maps in CML mapping, and scrambling was carried out at the bit level of the image. But either the fractional-order chaotic system or the CML system have periodic windows in the bifurcation diagrams which implies parameters can only generate local chaotic behavior. The parameters should be selected carefully for image encryption. In our paper, we mainly apply the 2DCML fractional-order chaotic system to the encryption process. In comparisons with the traditional logistic map and the fractional-order chaotic system, the 2DCML fractional-order chaotic system contains good features as larger key space, better chaotic ergodicity, less periodic windows in bifurcation diagrams, the larger range of parameters for chaotic behaviors. In addition, the value of chaotic sequences that generated by the 2DCML fractional-order chaotic system are larger than the traditional 2DCML system with the logistic mapping. Therefore, the proposed system is very suitable to image encryption.

The security of encryption algorithm is an important indicator to test whether the algorithm is reliable and efficient. Some studies show that chaotic encryption system will have security problems^21,36–39^. Xie et al.^37^ analyzed the steps of Fridrich’s algorithm with mathematical language, found out some defects in the application of Solak’s attack method, and provided some optimization basis for the attack scheme of Fridrich’s algorithm and derivative algorithm. Akhavan et al.^38^ pointed out that Eslami’s algorithm has security problems such as low resistance to selection attacks and the key space is not ideal. At the same time, the strategy of optimizing the image encryption algorithm was given. When improving the chaotic encryption system of Baptista-type cryptosystem, Chen et al.^36^ proposed that in order to ensure the security of encryption, it is necessary to ensure that the system does not reveal the chaotic state information during iteration, and the key space needs to be large enough to resist the attack of direct exhaustive search of the key. Wheeler et al.^39^ pointed out that there may be problems such as short period and strong dependence on specific values when using computer to realize chaotic mapping. In our paper the secret keys of the proposed algorithm include five parameters which the total key space is more than 2^425^, and it can resist exhaustive search attacks. The chaotic sequences generated by the 2DCML fractional-order chaotic system depend on the input plain image and the chaotic sequence will change completely if the input image changes, which provides the algorithm has stronger anti chosen plain text attack ability. In addition, we select special images (“White” and “Black”), binary image and color image to encrypt and simulation results show that the encryption effect is better. We have also completed tests of resisting noise attack and data loss, and the results show that the main information of images are still retained, so it shows that the algorithm has good ability to resist attacks. As described in “The new features of the proposed chaotic system in dynamical behaviors” section the 2DCML fractional-order chaotic system has better chaotic ergodicity in dynamical behaviors and both the parameter range and the values of chaotic sequences are larger. Moreover, the application of OF-LSTMS increases the complexity of the algorithm, so the encryption scheme will not leak the information of chaotic sequences. Therefore, our encryption algorithm has better security. Implementation speed is another important indicator to test whether the encryption system is efficient. In the process of image encryption, through the combination of chaotic sequences and OF-LSTMS, the proposed algorithm can change the pixel position while changing the pixel value, that is, permutation and diffusion are carried out at the same time, so the time consumption is less and the proposed encryption algorithm is efficient.

The rest of this paper is organized as follows. In “The application of OF-LSTMS in the proposed algorithm” and “The 2DCML fractional-order chaotic system” sections the OF-LSTMS and the 2DCML fractional-order chaotic system are presented respectively. The proposed image encryption and decryption scheme are described in “The proposed image encryption and decryption algorithm” section. Simulation results and performance analyses are reported in “Performance analyses” section.

## The application of OF-LSTMS in the proposed algorithm

**Figure 1:**
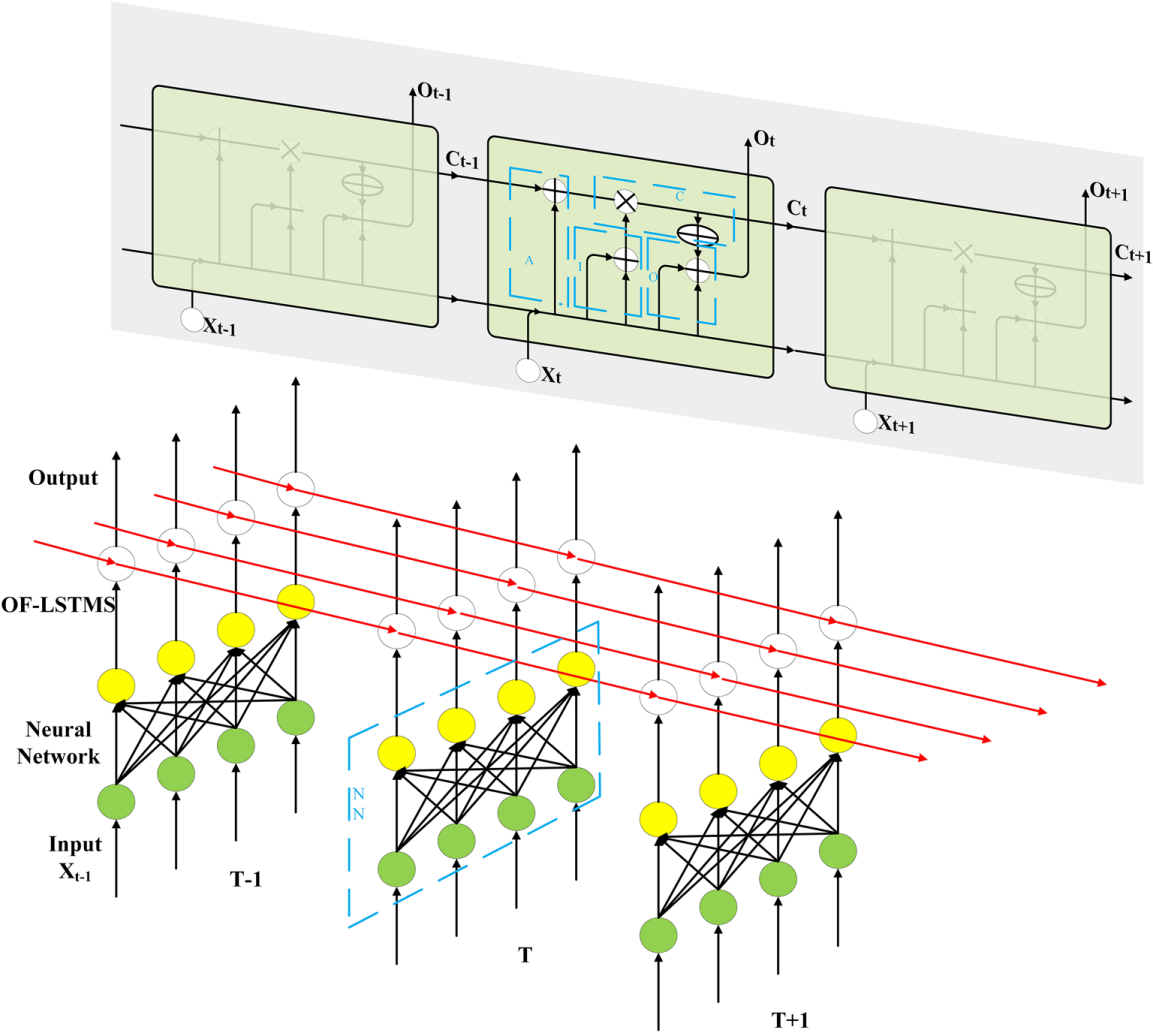
The OF-LSTMS.

LSTM structure is a chain structure with recurrent neural network module. It adopts matrix multiplication calculation method and gradient update parameter training method. LSTM structure consists of four parts: memory unit, forgetting gate, input gate and output gate^29,46–50^. Image encryption algorithm requires reversibility and does not need to learn features of the sequence, so it is necessary to modify the structure of LSTM. Based on the structure of LSTM, we propose the Once Forward Long Short Term Memory Structure (OF-LSTMS), which uses XOR operation instead of traditional matrix operation. The OF-LSTMS can realize that the encrypted image be obtained after only one forward propagation after original image entering the algorithm. The OF-LSTMS is as shown in Fig. 1.

**Figure 2:**
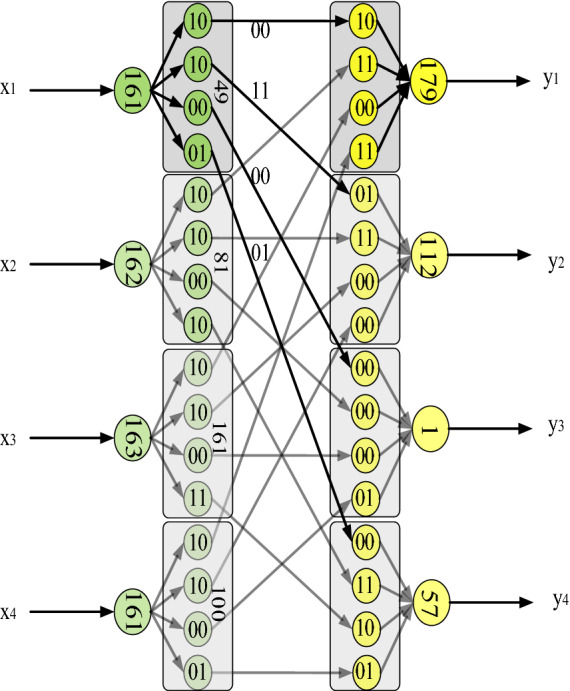
XOR operation of NN structure in the OF-LSTMS.

The operation used by the Neural Network (NN) structure of the traditional LSTM is matrix multiplication (MM), but it is not applicable in the image encryption task. MM cannot make the sum items (after being activated by MOD function) included in the output neurons be accurately restored to the input neurons during the decryption process, so the operation used by the NN structure of OF-LSTMS is XOR. The blue box NN in Fig. 1 (the part of The OF-LSTMS) shows the formal representation of NN structure and Fig. 2 shows the details of the XOR operation used by the NN structure of OF-LSTMS. As shown in Fig. 2, there are four original pixels, each of which contains 8-bit binary values. These pixels are divided into four groups of binary values, and XOR operation is performed with the same four sets of binary values (the values are from chaotic sequences). This operation process is called segmentation as shown in Fig. 2 (The part on the left which marked by green). In addition, the four groups of binary values in each rectangle in Fig. 2 come from the binary values of four different original pixels, which are finally calculated and then converted to decimal values. This operation process is called recombination as shown in Fig. 2 (The part on the right which marked by yellow).

The length of binary value used for recombination is 8 in order to ensure that no information is lost in the process of converting binary value to decimal value. Therefore, the number of neurons in the process of segmentation and recombination of the OF-LSTMS can only be multiple of 2, and the upper limit is 8. If the neurons and parameters are selected as 2 groups, 4 groups or 8 groups, it is necessary to select 2, 4 or 8 neurons as the neurons to enter the OF-LSTMS . We choose the number of neurons as 4 for balance the quality and efficiency of encryption.

In this paper, the mathematical formulas are shown in Eq. (1).

1 $$\left\{ {\begin{array}{*{20}l} {bitDic(X_{t} ) = \{ (X_{t} )_{12}^{bit} ,(X_{t} )_{34}^{bit} ,(X_{t} )_{56}^{bit} ,(X_{t} )_{78}^{bit} \}} \hfill \\ {NN(X_{t} ,CS_{t}^{X} )} = \{ bitDic(X_{t} )\;{\text{XOR}}\;bitDic(CS_{t}^{X})\} \hfill \\ {bitCon(x_{1}^{bit} ,x_{2}^{bit} ,x_{3}^{bit} ,x_{4}^{bit} ) = bin2dec([x_{1}^{bit} ,x_{2}^{bit} ,x_{3}^{bit} ,x_{4}^{bit} ])} \hfill \\ {X_{t}^{NN} = bitCon(NN(X_{t} ,CS_{t}^{X} ))\quad } \hfill \\ \end{array} } \right.,$$

where $bitDic(X_{t} )$ is a multivalued function that the binaries of the input $X_{t}$ divide into 4 parts and each part has 2 bits. $NN(X_{t} ,CS_{t}^{X} )$ is a mathematical formula for the NN structure of the OF-LSTMS that contains 4 neurons. $CS_{t}^{X}$(from the chaotic sequences) is the parameters of the corresponding inputs at the current moment. $bitCon(x_{1}^{bit} ,x_{2}^{bit} ,x_{3}^{bit} ,x_{4}^{bit} )$ is a function that combines 4 binaries of 2 bits into 8 bits and converts them to decimals. $X_{t}^{NN}$ is the outputs of the NN structure of the OF-LSTMS.

**Figure 3:**
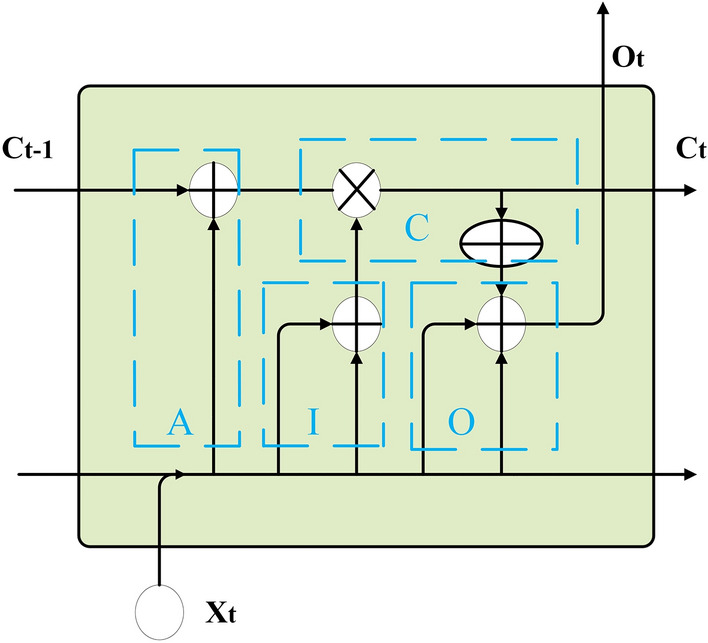
The part of the OF-LSTMS.

The function of the forgetting gate in the traditional LSTM is to determine which information inherited from the cell body at the previous moment to discard, which is determined by the input sequence at the current moment and the output at the previous moment. But it is not applicable in the image encryption task. The essence of the image encryption task is to scramble the image, and the less original image information contains in the encrypted image, the better the encryption effect. Therefore, the OF-LSTMS proposed in this paper designs the forgetting gate to the increasing gate, as shown in blue box A in Fig. 3. The cell body information at the current moment is obtained by accumulating with Eq. (2) the inputs’ parameters at the previous moment and the information inherited from the cell body at the previous moment chaotically.

2 $$C_{t}^{temp} = {\text{MOD}}(C_{t - 1} + CS_{t - 1}^{X} ,256),$$

where $C_{t - 1}$ is the information inherited from the cell body at the previous moment. $CS_{t - 1}^{X}$ is the inputs’ parameter at the previous moment. $C_{t}^{temp}$ is the information at the current moment after the increasing gate.

The function of the input gate in the traditional LSTM is to determine which information is added to the information at the current moment after the increasing gate, and it uses the multiplication operation. But the input gate in the OF-LSTMS uses add operation, which is due to the limitations in the image encryption. The input gate in the OF-LSTMS is shown in blue box I in Fig. 3 and can be represented by

3 $$\tilde{C}_{t}^{temp} = {\text{MOD}}(CS_{t - 1}^{X} + CS_{t}^{X} ,256),$$

where $\tilde{C}_{t}^{temp}$ is the information to be added to the information of the cell body at the current moment after the input gate.

The operation used in the OF-LSTMS is different from the operation used in traditional LSTM, and it uses XOR. As shown in blue box C in Fig. 3, it can be represented by

4 $$C_{t} = C_{t}^{temp} {\text{XOR}}\tilde{C}_{t}^{temp} ,$$

where $C_{t}$ is the information of the memory unit in the cell body that will be used to encrypt the input sequence at the current moment.

The function of the output gate in the OF-LSTMS is to use the information in the cell body and additional chaotic sequences to encrypt the input sequence. It is shown in blue box O in Fig. 3, and can be represented by

5 $$\left\{ {\begin{array}{*{20}l} {C_{t}^{O} = {\text{MOD}}(C_{t} + CS_{t}^{C} ,256)} \hfill \\ {X_{t}^{temp} = {\text{MOD}}(X_{T}^{NN} + CS_{t}^{O} ,256)} \hfill \\ {O_{t} = {\text{MOD}}(X_{t}^{temp} + C_{t}^{O} ,256)} \hfill \\ \end{array} } \right.,$$

where $CS_{t}^{C}$ and $CS_{t}^{O}$ (from the chaotic sequence) are the chaotically information added to the information in the cell body and input sequence at the current moment. $C_{t}^{O}$ and $X_{t}^{temp}$ are the information after $C_{t}$ and $X_{t}^{NN}$ are chaotic. $O_{t}$ are the output, which are the sum of $C_{t}^{O}$ and $X_{t}^{temp}$.

In this algorithm, the original image sequence enters into the OF-LSTMS in order to operate with different chaotic sequences. The memory information *C*_*t*_ and the encrypted pixel value *O*_*t*_ can be obtained at the same time. According to the memory information *C*_*t*_, the new position of the encrypted pixel can be obtained, that is, the encrypted pixel position and pixel value are determined at the same time. The synchronization of permutation and diffusion can improve the efficiency of encryption.

## The 2DCML fractional-order chaotic system

### The proposed chaotic system

Comparison with the traditional logistic map, the fractional-order logistic map contains larger key space and more parameters. Zhang et al.^52^ exhibited the features of the fractional-order chaotic system in dynamical behaviors. The following iteration equation is obtained^52^:

6 $$x_{n + 1} = x_{n} + \mu x_{n} (1 - x_{n} )\frac{{r^{\alpha } }}{\Gamma (\alpha + 1)}.$$

The parameters *α*, *μ*, *r* and the initial values *x*_0_ of the fractional-order logistic system can be designed the secret keys.

Based on the fractional-order logistic map, the proposed system coupled by the neighborhood links of the 2DCML system^53^ as follows:

7 $$x_{n + 1} (i,j) = (1 - \varepsilon )f[x_{n} (i,j)] + \frac{\varepsilon }{4}\left\{ {f[x_{n} (i - 1,j)] + f[x_{n} (i + 1,j)] + f[x_{n} (i,j - 1)] + f[x_{n} (i,j + 1)]} \right\}$$

where *i* , *j* are the lattices ($1 \le i,j \le L$) , *ε* is the coupling parameter ($0 \le \varepsilon \le 1$), *n* is the time index (*n* = 1,2,3, …) and $f(x)$ is the fractional-order logistic map with the iteration equation is obtained as Eq. (6).

### The new features of the proposed chaotic system in dynamical behaviors

To qualify the new features of the proposed system in dynamical behaviors mathematically, the bifurcation diagrams, the Lyapunov exponents, the space-amplitude diagrams and the patterns diagrams are widely analyzed theoretically in this section.

**Figure 4:**
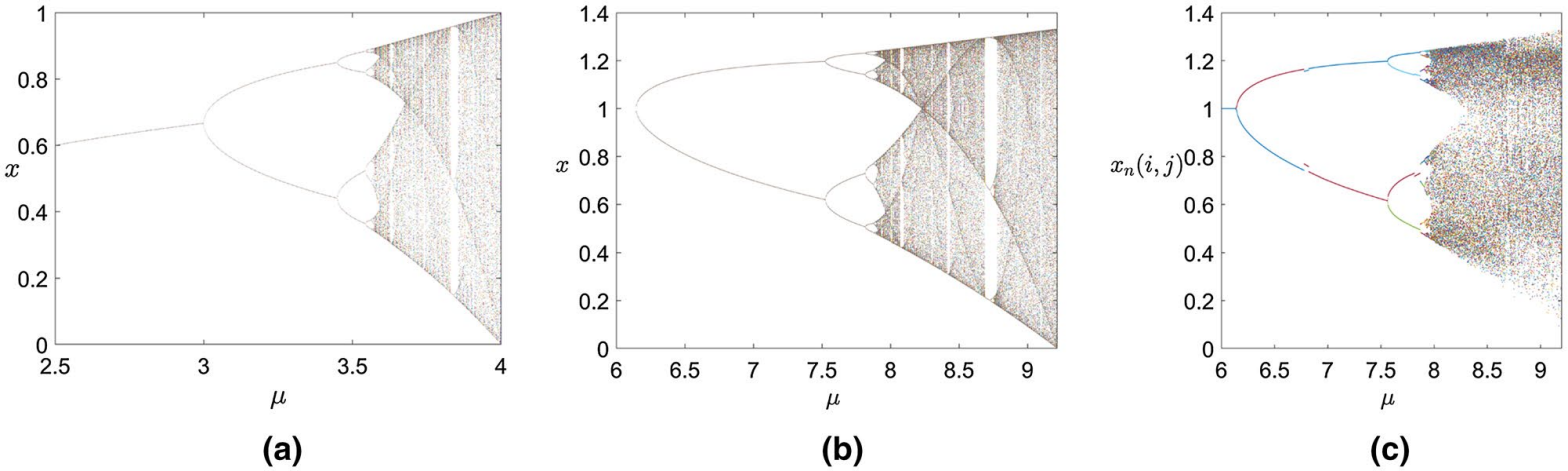
Bifurcation diagrams. (**a**) The traditional logistic chaotic system^52^, (**b**) the fractional-order chaotic logistic system with $\alpha = 0.85,\;r = 0.25$^52^, (**c**) the 2DCML fractional-order chaotic system with $\alpha = 0.85,\;r = 0.25,\;\varepsilon = 0.39$.

In the proposed system, the bifurcation diagrams are shown in Fig. 4c. In Fig. 4a the parameter *μ* in the traditional logistic map is (3.57, 4). In Fig. 4b the parameter *μ* in the fractional-order logistic differential map breaks the range of $\mu \in (3.57,4)$ and the numerical range of chaotic sequence *x*_*n*_ also breaks the range of (0,1). While an important phenomenon in Fig. 4c is that the proposed system not only retains the advantages of the fractional-order logistic map, but also its periodic windows are reduced substantially compared with the Fig. 4a,b and the gaps between bifurcation points vary closer. Due to the neighborhood coupling leading the instability of the possible periods of orbits, the times of period doubling bifurcations is misled and unobvious. Therefore, both the parameter range and the values of chaotic sequences of the proposed system are larger than the traditional logistic mapping and the fractional-order chaotic logistic system. In our encryption algorithm, the proposed chaotic system is selected to generate chaotic sequences, and the parameters are selected as secret keys.

**Figure 5:**
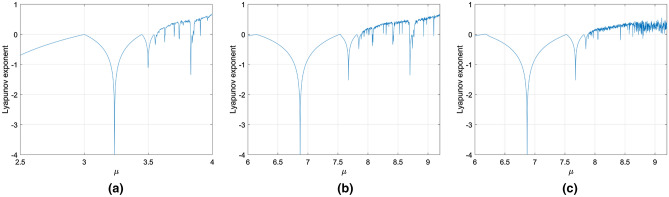
Lyapunov exponent curves. (**a**) The traditional logistic chaotic system^52^, (**b**) the fractional-order chaotic logistic system with $\alpha = 0.85,\;r = 0.25$^52^, (**c**) the 2DCML fractional-order chaotic system with $\alpha = 0.85,\;\;r = 0.25,\;\;\varepsilon { = 0}{\text{.39}}$.

Any system holding chaotic behavior presented at least one positive Lyapunov exponent. In Fig. 5, comparing with the Lyapunov exponents of the fractional-order chaotic logistic system and the traditional logistic maps system, the positive interval of Lyapunov exponents of the proposed chaotic system is far greater than that of the former two systems. Therefore, the proposed chaotic system has strong chaotic characteristics and can generate better chaotic sequences. It is more suitable for encryption algorithm.

**Figure 6:**
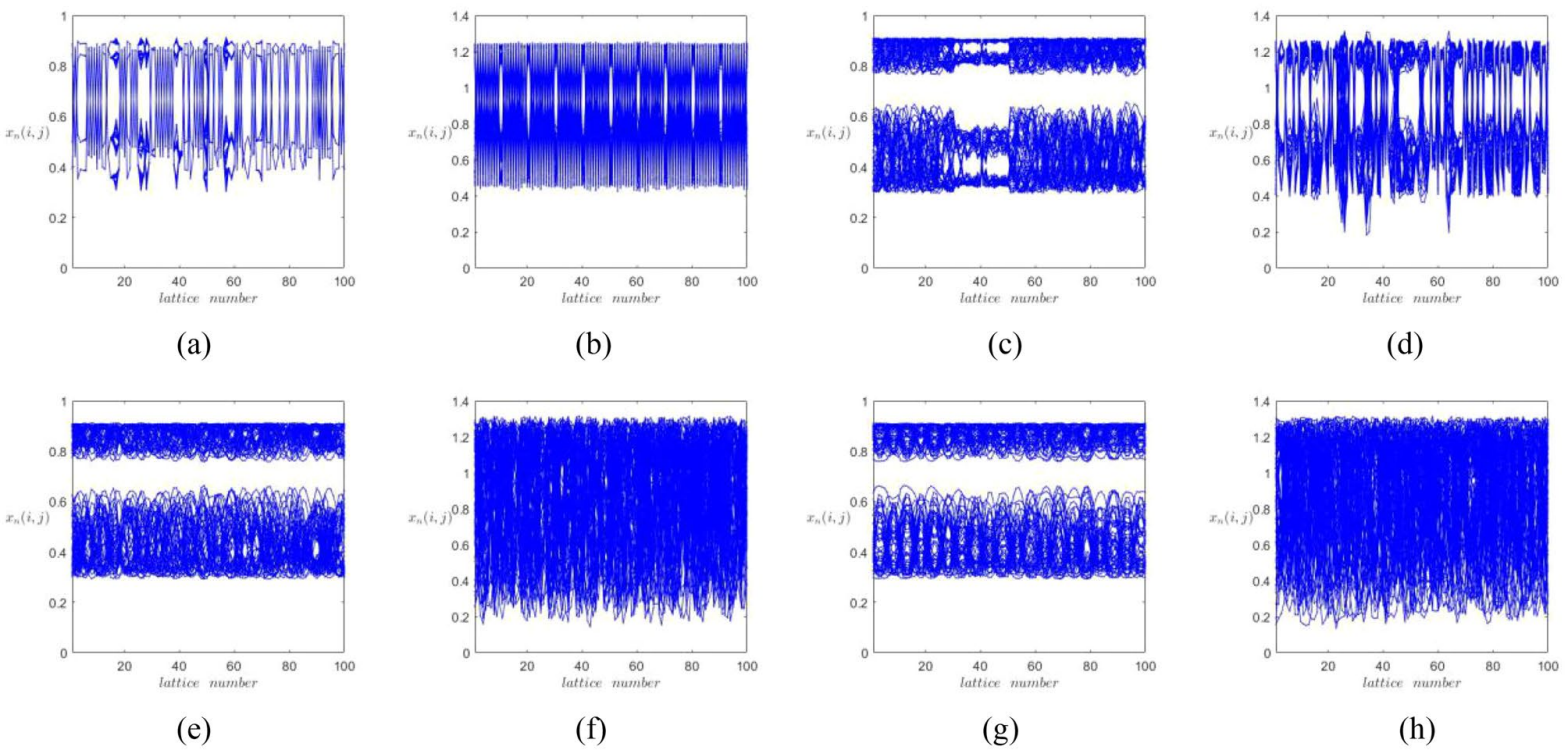
The space-amplitude plots when times = 1000. (**a**) the 2DCML system (*ε* = 0.1), (**b**) the proposed system (*ε* = 0.1), (**c**) the 2DCML system (*ε* = 0.3), (**d**) the proposed system (*ε* = 0.3), (**e**) the 2DCML system (*ε* = 0.5), (**f**) the proposed system (*ε* = 0.5), (**g**) the 2DCML system (*ε* = 0.7), (**h**) the proposed system (*ε* = 0.7).

**Figure 7:**
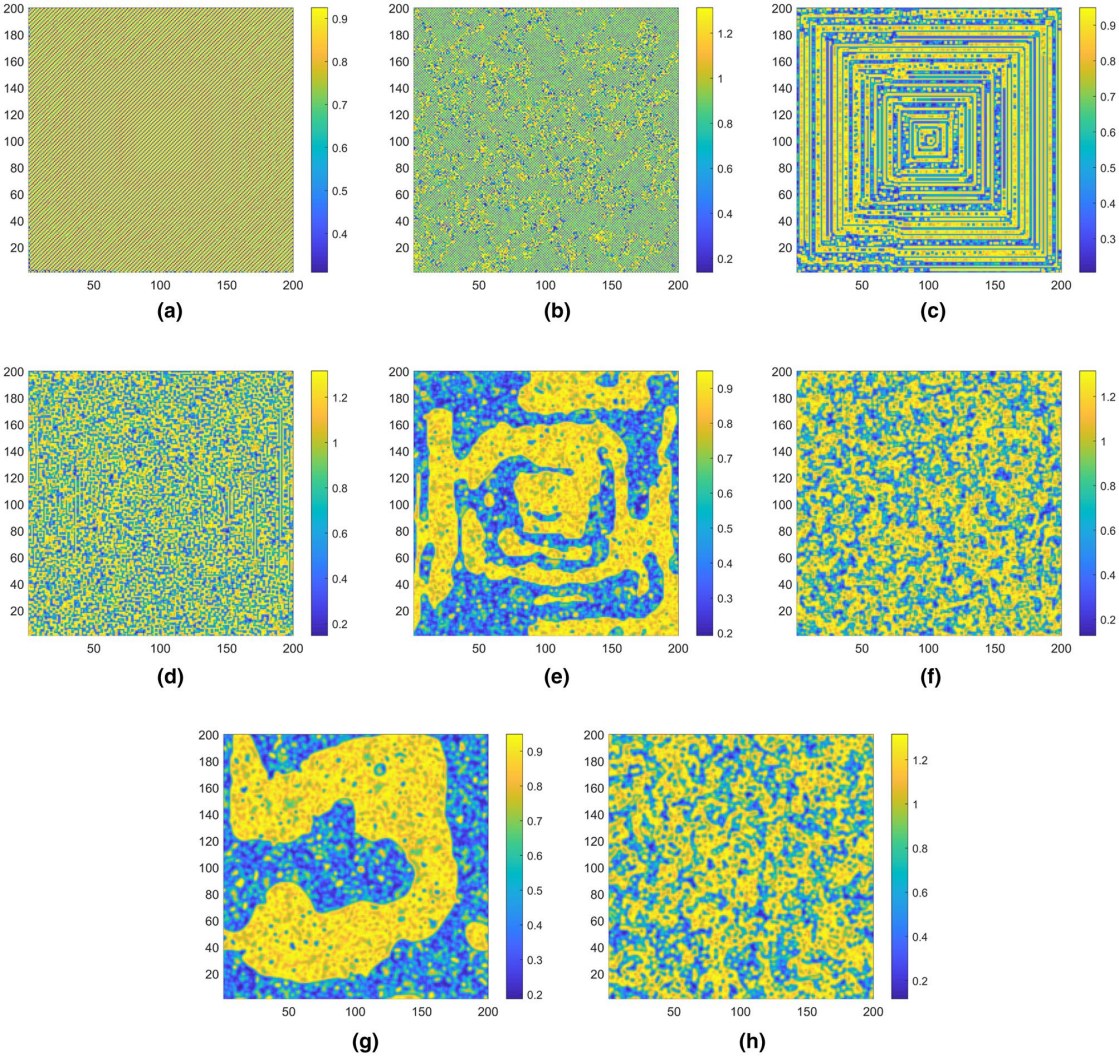
Snapshot patterns when times = 1000. (**a**) the 2DCML system (*ε* = 0.1), (**b**) the proposed system (*ε* = 0.1), (**c**) the 2DCML system (*ε* = 0.3), (**d**) the proposed system (*ε* = 0.3), (**e**) the 2DCML system (*ε* = 0.5), (**f**) the proposed system (*ε* = 0.5), (**g**) the 2DCML system (*ε* = 0.7), (**h**) the proposed system (*ε* = 0.7).

Because the fractional-order logistic map contains larger key space and more parameters comparing with the traditional logistic map, the proposed system contains more universality of chaos in space than the 2DCML system with the same parameter *ε*, which is shown in the space-amplitude plots as Fig. 6. In addition, the snapshot patterns shown in Fig. 7 indicate that the proposed system presents more complex chaotic resolutions than the 2DCML system. For example, Fig. 7f indicates that the same parameter *ε* which lead the proposed system in fully developed turbulence pattern, can only lead the 2DCML system in defect turbulence pattern which is shown in Fig. 7e.

## The proposed image encryption and decryption algorithm

Without loss of generality, the images are employed to present the encryption scheme for simplicity. The corresponding encryption algorithm and decryption algorithm can be presented as follows.

### Encryption algorithm

Step 1.: Generate the key sequence *K* and the initial values $\alpha ^{\prime}$, $\mu ^{\prime}$, $x^{\prime}$, $r^{\prime}$ and $\varepsilon ^{\prime}$ of the proposed system. The proposed algorithm utilizes a 160-bit secret key *K*, which is generated by the hash algorithm MD2. For source images, even if only one bit is changed, its hash value will change completely. By dividing the 160-bit secret key into 16-bit blocks (*K*_*i*_), and the new initial values can be obtained by the following formulas:
  $$\alpha ^{\prime} = \alpha + (bin2dec(k_{1} \oplus k_{2} )) \times 10^{ - 6} ,$$
  $$\mu ^{\prime} = \mu + (bin2dec(k_{3} \oplus k_{4} )) \times 10^{ - 6} ,$$
  $$x^{\prime} = x_{0} + (bin2dec(k_{5} \oplus k_{6} ) \times 10^{ - 6} ,$$
  $$r^{\prime} = r + (bin2dec(k_{7} \oplus k_{8} )) \times 10^{ - 6} ,$$
  $$\varepsilon ^{\prime} = \varepsilon + (bin2dec(k_{{9}} \oplus k_{{{10}}} )) \times 10^{ - 6}$$
  where $\alpha$, $\mu$, $x_{{0}}$, $r$ and $\varepsilon$ are the initial given values.
Step 2.: The chaotic sequences are iterated according to Eq. (7), and the chaotic sequences used in the OF-LSTMS are determined.
Step 3.: The original image is transformed into a sequence, and each 4 pixels value is regarded as a subsequence.
Step 4.: The subsequences are encrypted in the OF-LSTMS according to “The application of OF-LSTMS in the proposed algorithm” section. Assume the size of the encrypted image is 512 × 512 and the image is divided into 65,536 pixel sequences with 4 pixels as a group. Then the shape of input data into the OF-LSTMS is [1, 65,536, 4].

**Figure 8:**
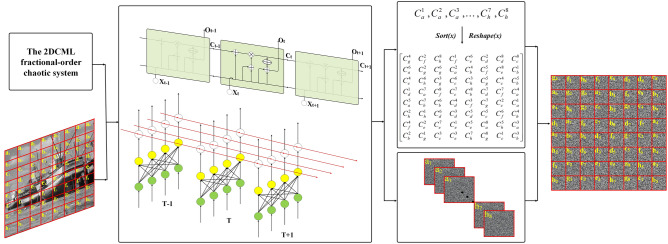
The encryption process.

Finally, the ciphered image is obtained. The encryption process is shown as Fig. 8.

### Decryption algorithm

**Figure 9:**
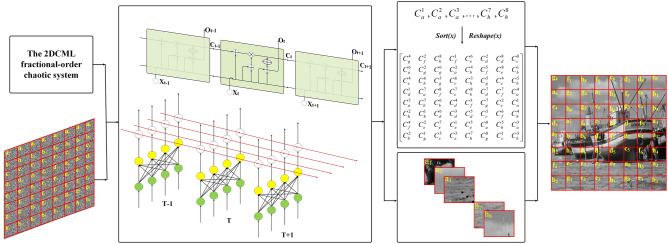
The decryption process.

The decryption process is contrary to the encryption process. Using the secret keys provided by the sender, the receivers decrypt the cipher image according to the contrary operations of the encryption algorithm. The decryption process is shown as Fig. 9.

**Figure 10:**
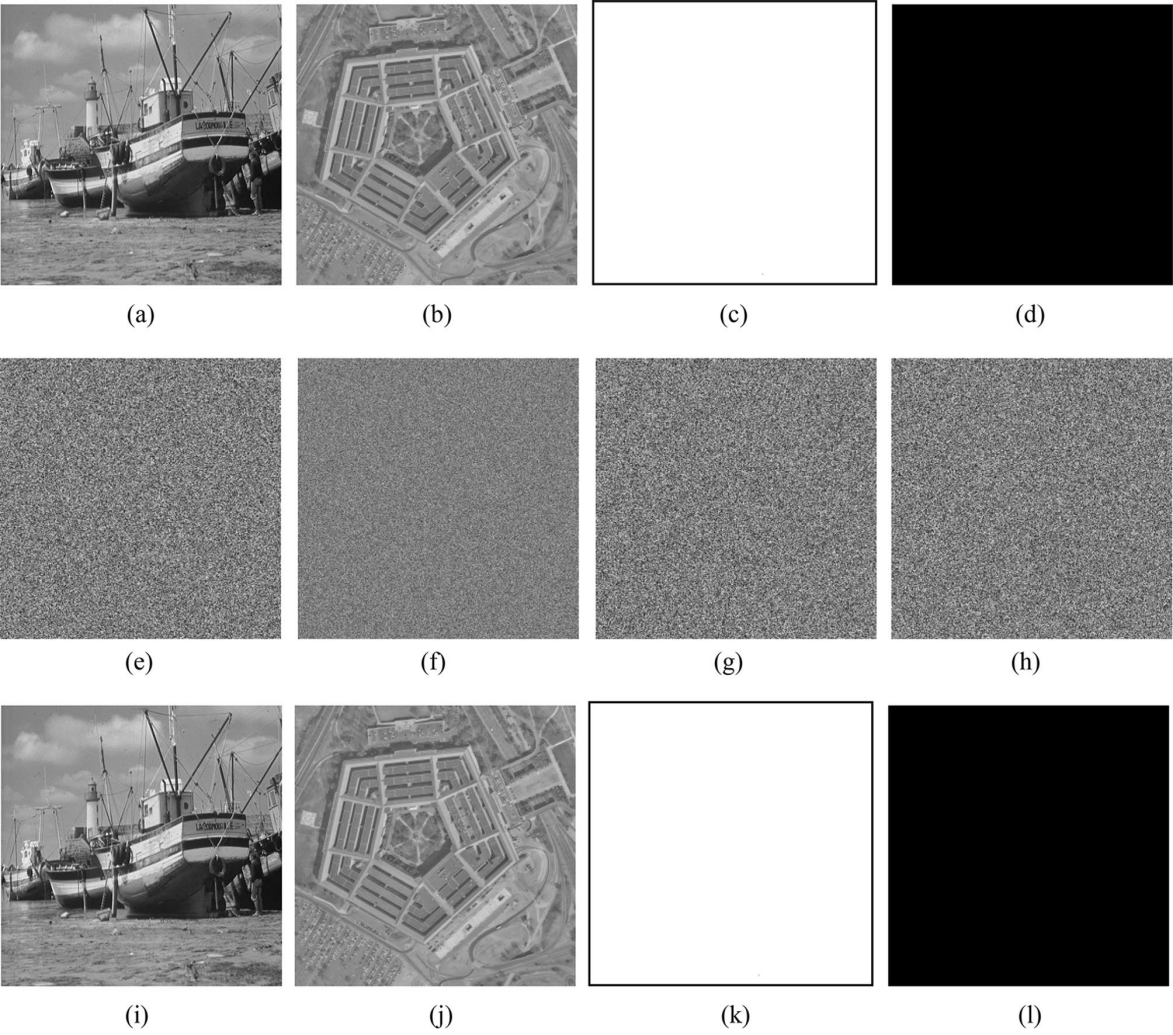
Encryption and decryption of images. (**a**) Original image of Boat.512, (**b**) original image of image 3.2.25, (**c**) original image of White, (**d**) original image of Black, (**e**) encrypted image of Boat.512, (**f**) encrypted image of image 3.2.25, (**g**) encrypted image of White, (**h**) encrypted image of Black, (**i**) decrypted image of Boat.512. (**j**) decrypted image of image 3.2.25, (**k**) decrypted image of White, (**l**) decrypted image of Black.

**Figure 11:**
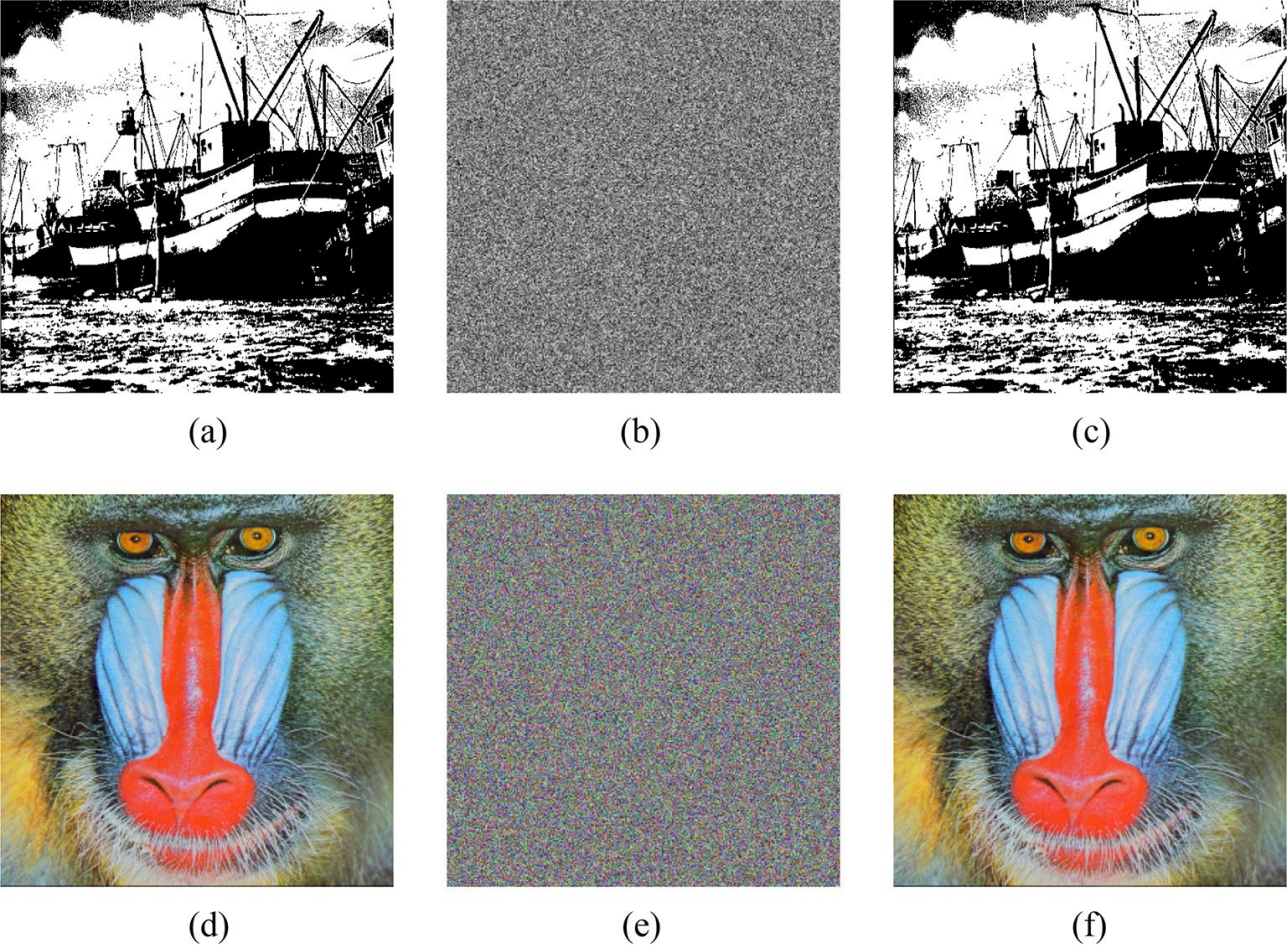
Encryption and decryption of binary image and color image. (**a**) Original image of Binary_boat.512, (**b**) encrypted image of Binary_boat.512, (**c**) decrypted image of Binary_boat.512. (**d**) original image of image 4.2.03, (**e**) encrypted image of image 4.2.03, (**f**) decrypted image of image 4.2.03.

In the experiments, the test images are the “Boat.512 (512 × 512)” , “image 3.2.25 (1024 × 1024) ” and color image “image 4.2.03 (512 × 512)” from the USC-SIPI image database. We also chose two special images “White (512 × 512)”, “Black (512 × 512)” and binary image “Binary_boat.512 (512 × 512)” to be encrypted. Figures 10 and 11 show the encryption and decryption of images for one round.

## Performance analyses

In order to evaluate the security of the proposed encryption algorithm, we undertake a series of statistical analysis on the encryption and decryption results, and show the analysis results in detail in this section.

### Key space

Only if the key space is large enough, it can resist violent attacks. The secret keys include decimal parameters *α*, *μ*, *r, ε* and the initial value *x*_0_. The total key space is 10^80^ if the accuracy of the computer is 10^16^. In the proposed encryption algorithm, because the total key space is more than 2^425^ that the key space can satisfy the security requirements.

### Key sensitivity

**Figure 12:**
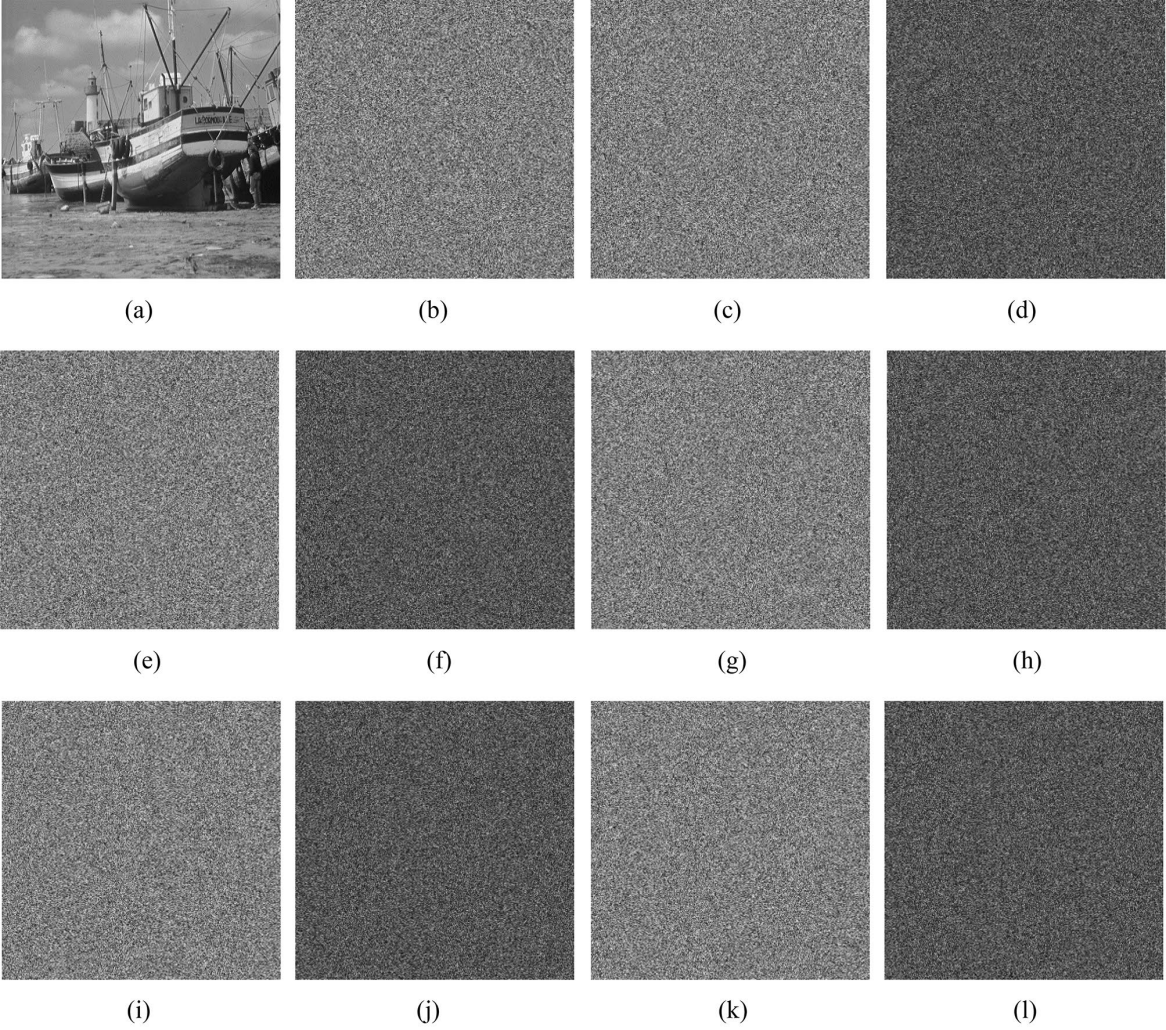
Keys sensitivity. (**a**) Original image of Boat.512, (**b**) encrypted image of Boat.512 using original *μ* = 9, *α* = 0.85, *r* = 0.25, *x*_0_ = 0.30565487923280 and *ε* = 0.39, (**c**) encrypted image of Boat.512 using changed *μ* = 9.0000000001, (**d**) difference between (**b**) and (**c**). (**e**) encrypted image of Boat.512 using changed *α* = 0.8500000001, (**f**) difference between (**b**) and (**e**). (**g**) encrypted image of Boat.512 using changed *r* = 0.2500000001, (**h**) difference between (**b**) and (**g**). (**i**) encrypted image of Boat.512 using changed *x*_0_ = 0.30565487923281, (**j**) Difference between (**b**) and (**i**), (**k**) encrypted image of Boat.512 using changed *ε* = 0.3900000001, (**l**) difference between (**b**) and (**k**).

The characteristic of chaotic system is that the small change of initial value will lead to completely different chaotic sequences. We modify one of the parameter values, while the others remain unchanged. Simulation and analysis show that small changes in the key will lead to significant changes in the output, so the algorithm is very sensitive to the key. Figure 12 shows the results of the corresponding *μ*, *α*, *r*, *x*_0_ and *ε* tests, respectively.

### Histogram analysis

**Figure 13:**
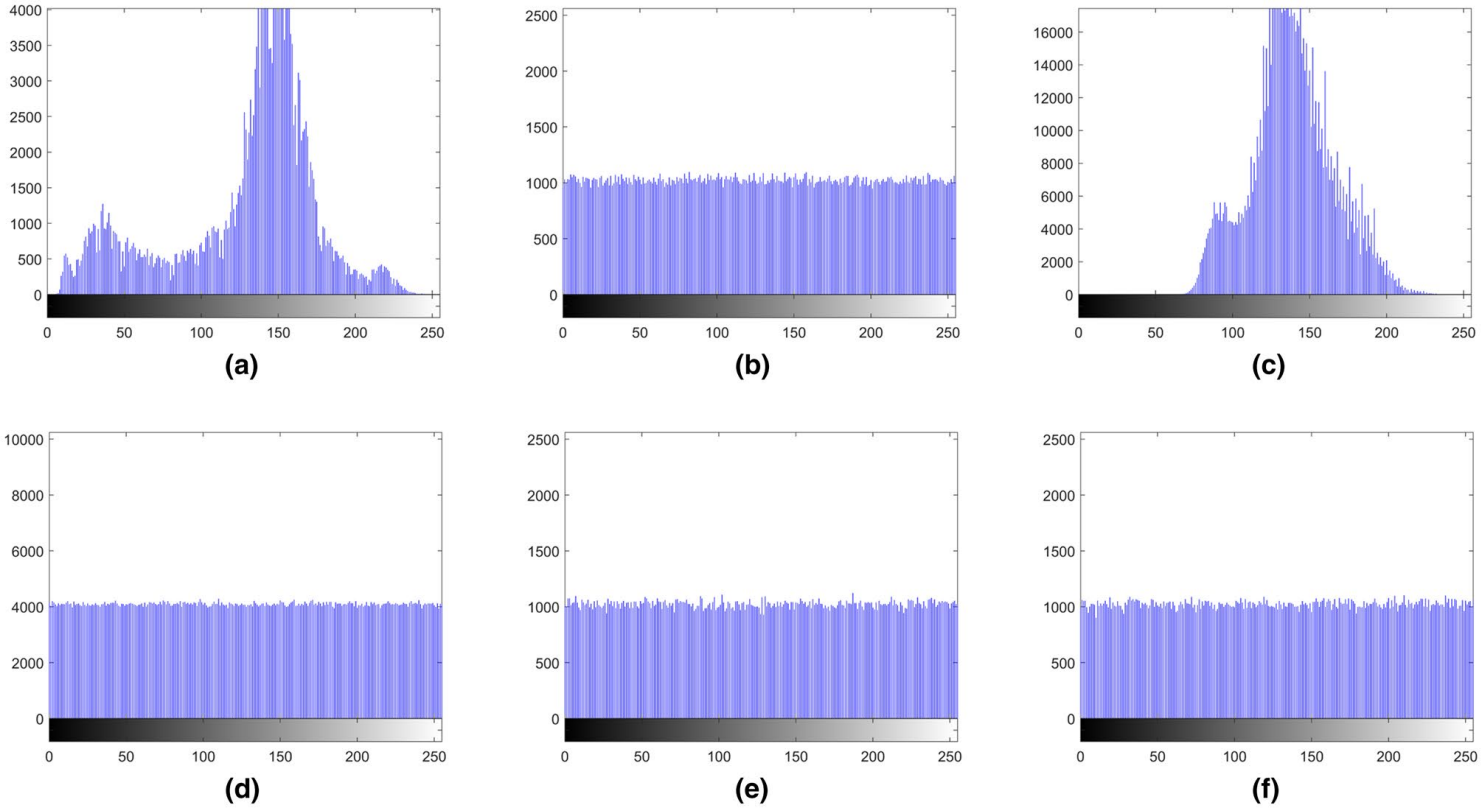
The histograms of gray images and encrypted images. (**a**) Histogram of Boat.512, (**b**) histogram of encrypted Boat.512, (**c**) histogram of image 3.2.25, (**d**) histogram of encrypted image 3.2.25, (**e**) histogram of encrypted White, (**f**) histogram of encrypted Black.

**Figure 14:**
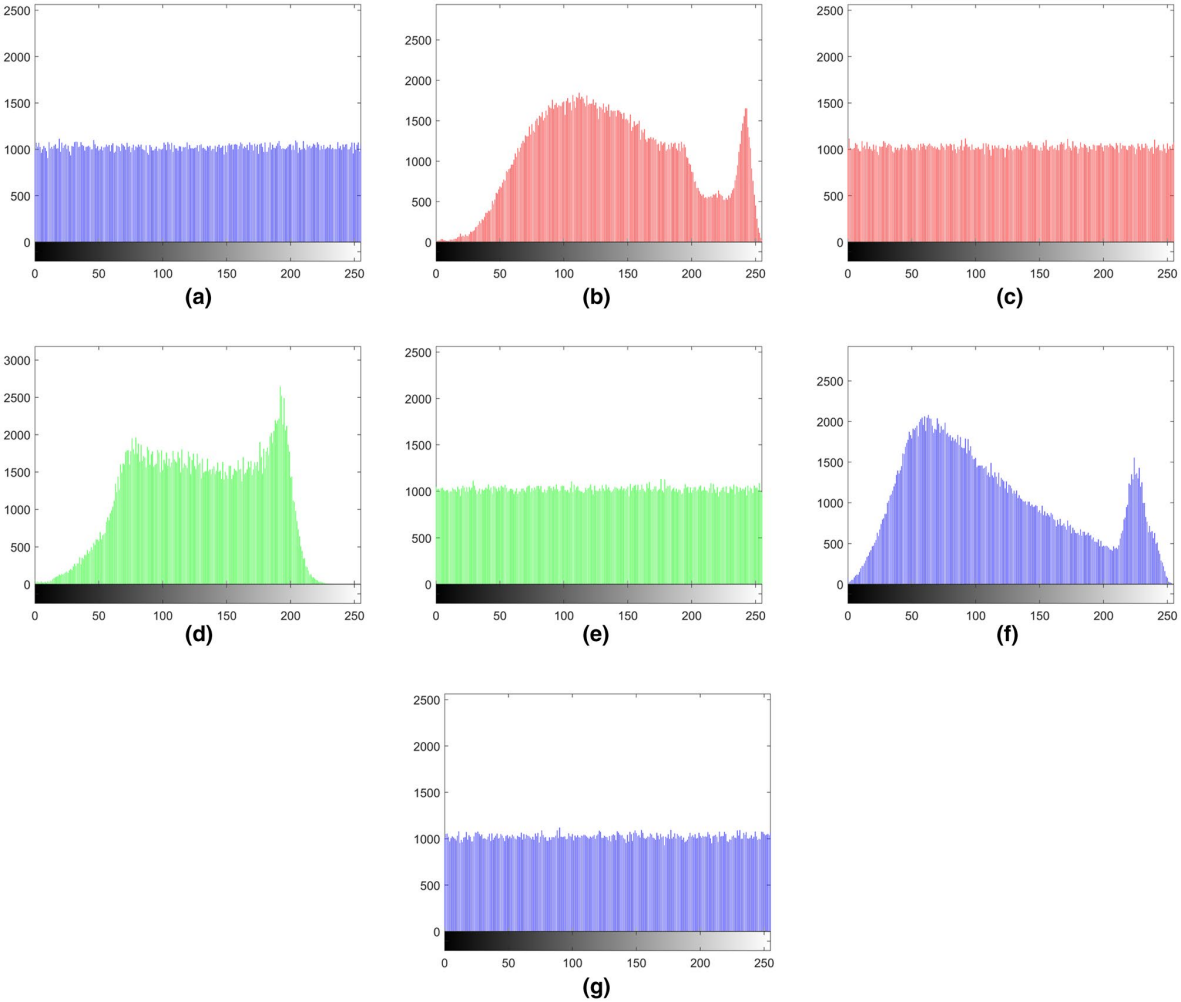
The histograms of the encrypted binary image, color image and encrypted color image. (**a**) Histogram of encrypted Binary_boat.512, (**b**) histogram of R channel of color image 4.2.03, (**c**) histogram of R channel of encrypted color image 4.2.03, (**d**) histogram of G channel of color image 4.2.03, (**e**) histogram of G channel of encrypted color image 4.2.03, (**f**) histogram of B channel of color image 4.2.03, (**g**) histogram of B channel of encrypted color image 4.2.03.

Histogram analysis is an important image analysis method, which can reflect the frequency distribution of pixel values in the image. Figures 13 and 14 show that encrypted images have completely different histograms against the original images. It shows that the encrypted image has no relationship with the original image. Therefore, the proposed image encryption algorithm can resist histogram analysis attacks.

### Differential attack

To evaluate the encryption algorithm’s ability to resist differential attacks, we employ the unified average changing intensity (UACI) and the number of pixels change rate (NPCR) which are defined by

8 $$UACI = \frac{1}{M \times N}\left[ {\sum\nolimits_{ij} {\frac{{|c_{1} (i,j) - c_{2} (i,j)|}}{255}} } \right] \times 100\% ,$$

9 $$\left\{ {\begin{array}{*{20}l} {D(i,j) = \left\{ {\begin{array}{*{20}l} {1, \;\;\; c_{1} (i,j) \ne c_{2} (i,j)} \\ {0, \;\;\; otherwise} \\ \end{array} } \right.} \\ {NPCR = \frac{{\sum\nolimits_{ij} {D(i,j)} }}{M \times N} \times 100\% } \\ \end{array} } \right.,$$

where *c*_1_and *c*_2_ are the two ciphered images. The test images also include some images from the USC-SIPI image database. The NPCR and UACI values about ciphered images are listed in Table 1, which shows that the proposed encryption algorithm is very sensitive to a pixel change in the original image.

**Table 1:** NPCR and UACI performance of ciphered images.

Images	NPCR (%)	UACI (%)
Baboon	99.59793	33.54939
Boat.512	99.61586	33.44338
Image 5.2.09	99.62120	33.43819
Image 5.3.02	99.60737	33.47900
Image 3.2.25	99.61567	33.46659
Image 7.2.01	99.60212	33.47211
White	99.60442	33.54164
Black	99.61929	33.53024
Average (gray images)	99.61048	33.49007
Binary_boat.512	99.62463	33.48465
Color image 4.2.03 (R-component)	99.61739	33.51193
Color image 4.2.03 (G-component)	99.61700	33.47095
Color image 4.2.03 (B-component)	99.63417	33.56694

Mean Squared Error (MSE) and Peak Signal to Noise Ratio (PSNR) can be used to test the efficiency of encryption and decryption^40,41^. We calculate the PSNR and the MSE of the original images and the encrypted images. It can be seen from Table 2 that the PSNR value is lower, while the MSE value is higher, which indicates that the image encryption process is more efficient. Similarly, we also calculate the PSNR and the MSE of the original images and the decrypted images. The results in Table 2 show that the PSNR value is higher and the MSE value is lower, indicating that the image decryption of algorithm is efficient too.

**Table 2:** PSNR and MSE.

Images	Encryption efficiency		Decryption efficiency	
	PSNR	MSE	PSNR	MSE
Baboon	9.8067	6.7985E+03	79.6917	6.9809E−04
Boat.512	9.2968	7.6455E+03	81.9820	4.1199E−04
Image 5.2.09	8.2124	9.8140E+03	86.2956	1.5259E−04
Image 5.3.02	8.7352	8.7008E+03	84.4098	2.3556E−04
Image 3.2.25	10.1299	6.3108E+03	97.9229	1.0490E−05
Image 7.2.01	6.3287	1.5143E+04	85.9313	1.6594E−04
White	4.7607	2.1728E+04	79.2524	1.5411E−04
Black	4.7503	2.1780E+04	80.9490	5.2261E−04
Average (gray images)	7.7526	1.2240E+04	84.5543	2.9392E−04
Binary_boat.512	4.7824	2.1619E+04	96.2956	1.5259E−05
Color image 4.2.03 (R-component)	8.7663	8.6387E+03	80.3296	6.0272E−04
Color image 4.2.03 (G-component)	9.2577	7.7145E+03	81.7471	4.3488E−04
Color image 4.2.03 (B-component)	8.3694	9.4654E+03	82.9210	3.3188E−04

### Correlation analysis

**Figure 15:**
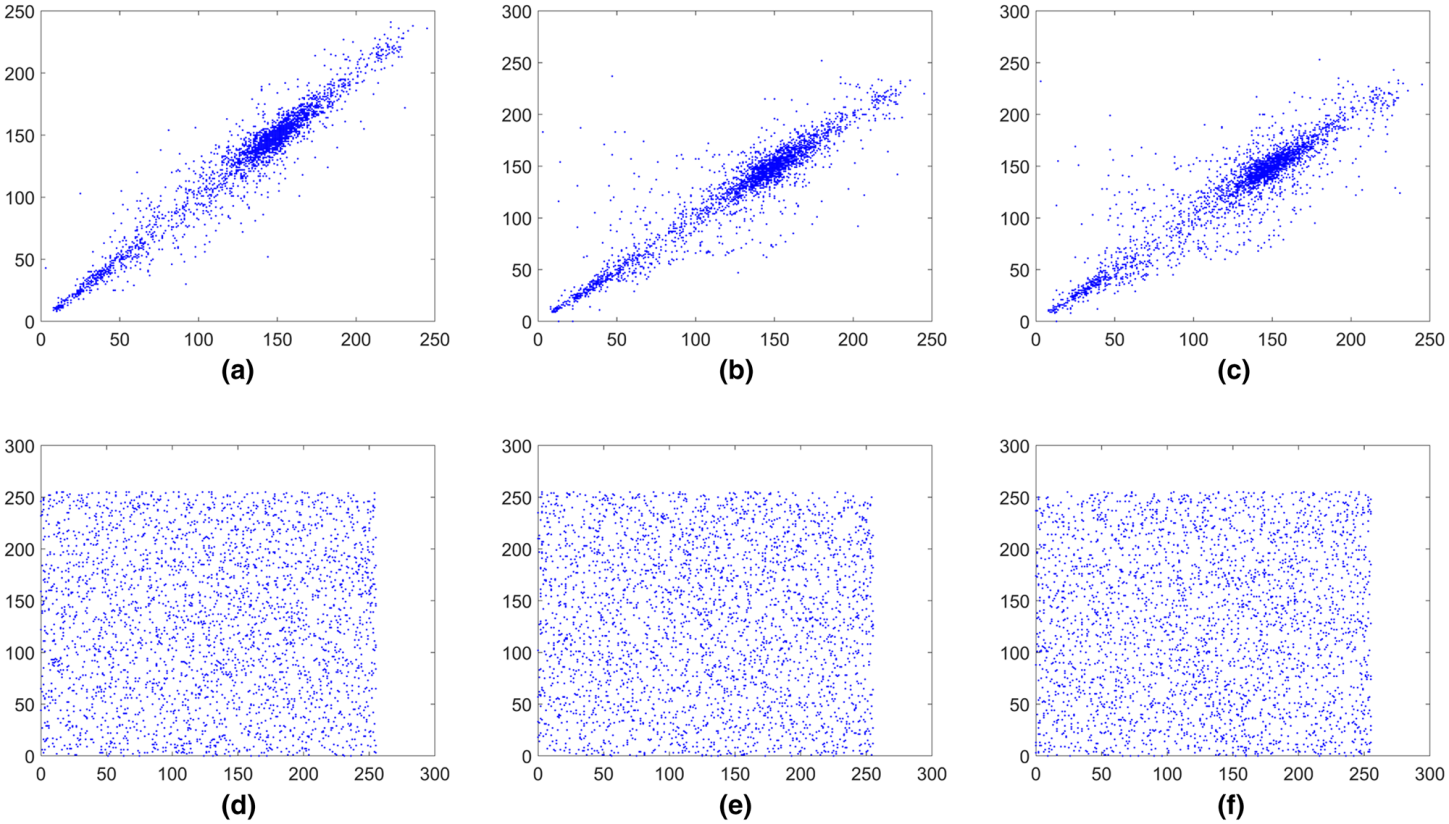
Distribution of adjacent pixels of the image Boat.512 and its encrypted images. (**a**) Horizontal direction of the image, (**b**) vertical direction of the image, (**c**) diagonal direction of the image, (**d**) horizontal direction of the encrypted image, (**e**) vertical direction of the encrypted image, (**f**) diagonal direction of the encrypted image.

**Figure 16:**
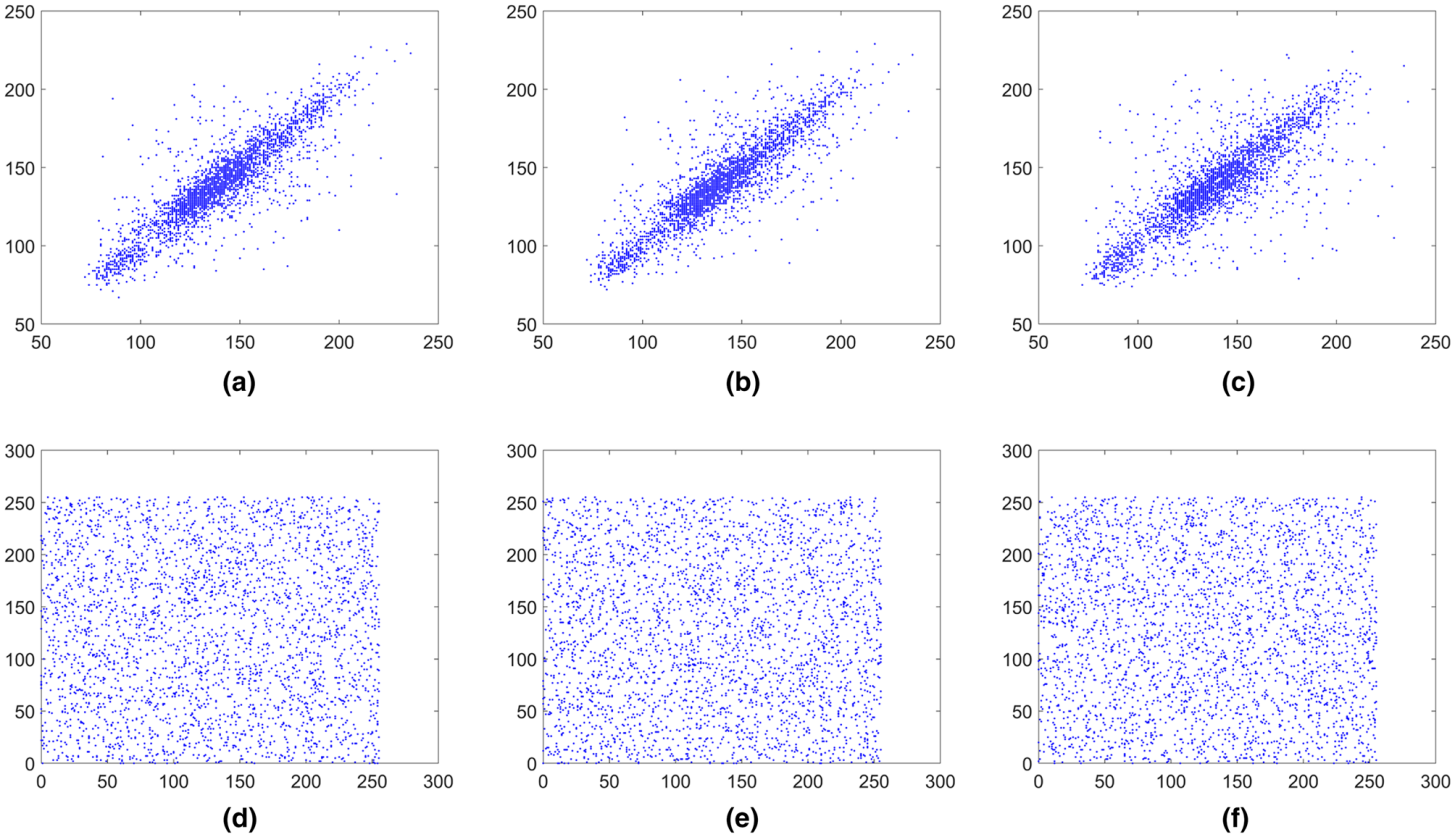
Distribution of adjacent pixels of the image 3.2.25 and its encrypted images. (a) Horizontal direction of the image, (**b**) vertical direction of the image, (**c**) diagonal direction of the image, (**d**) horizontal direction of the encrypted image, (**e**) vertical direction of the encrypted image, (**f**) diagonal direction of the encrypted image.

**Figure 17:**
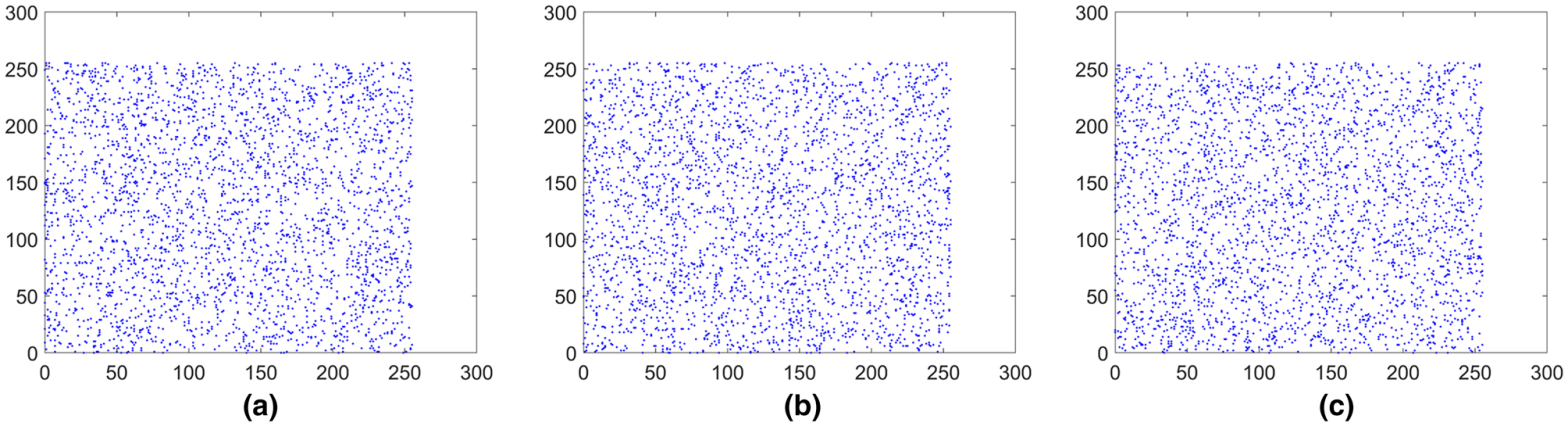
Distribution of adjacent pixels of the encrypted White. (**a**) Horizontal direction of the encrypted image, (**b**) vertical direction of the encrypted image, (**c**) diagonal direction of the encrypted image.

**Figure 18:**
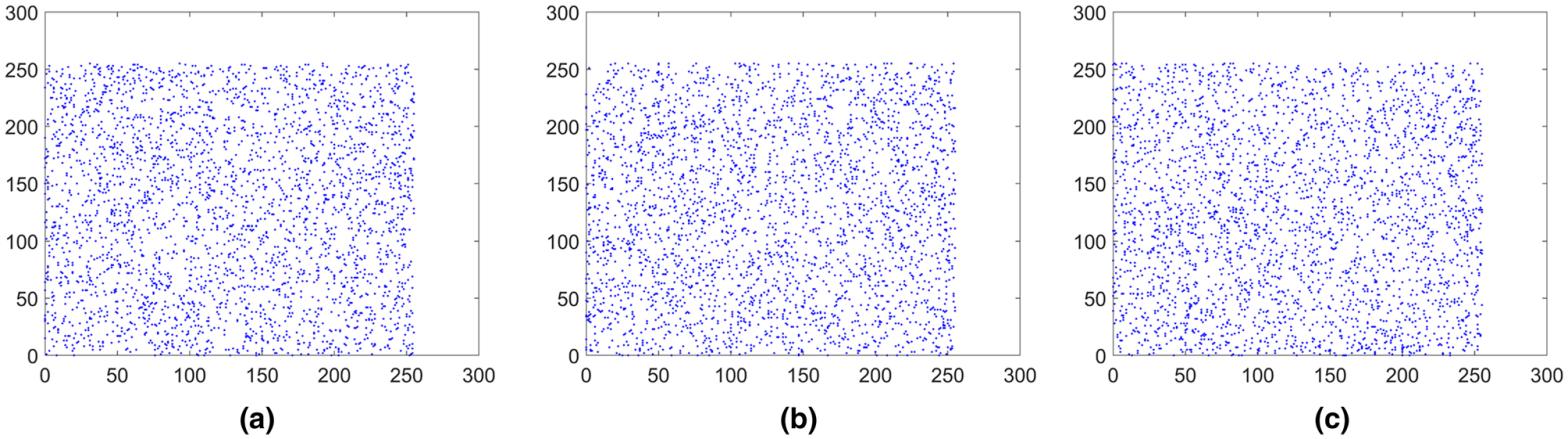
Distribution of adjacent pixels of the encrypted Black. (**a**) Horizontal direction of the encrypted image, (**b**) vertical direction of the encrypted image, (c) diagonal direction of the encrypted image.

**Figure 19:**
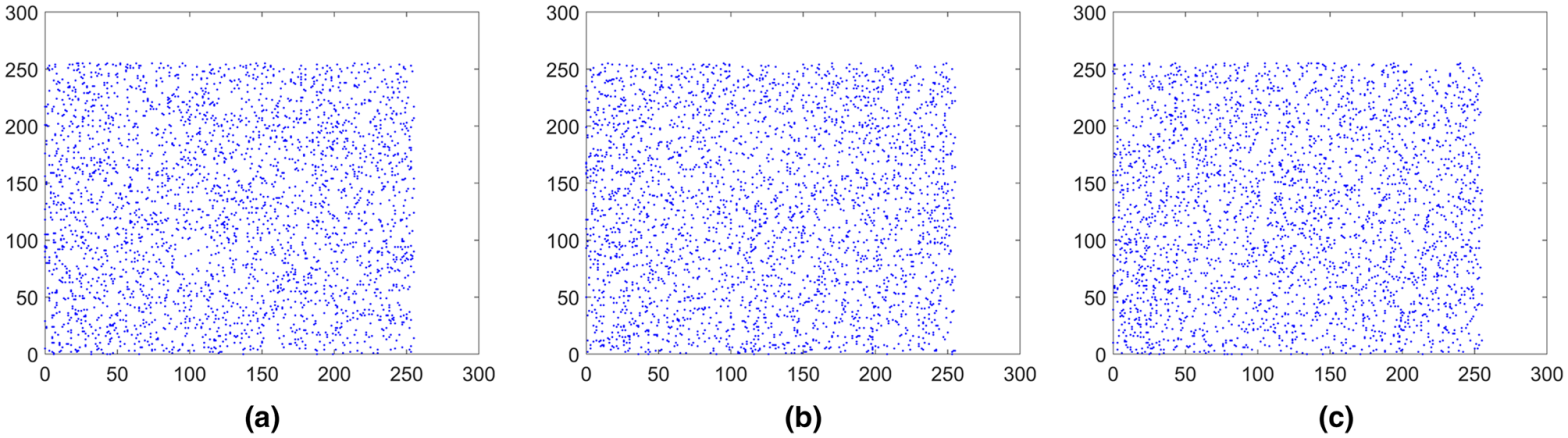
Distribution of adjacent pixels of the encrypted Binary_boat.512. (**a**) Horizontal direction of the encrypted image, (**b**) vertical direction of the encrypted image, (**c**) diagonal direction of the encrypted image.

**Figure 20:**
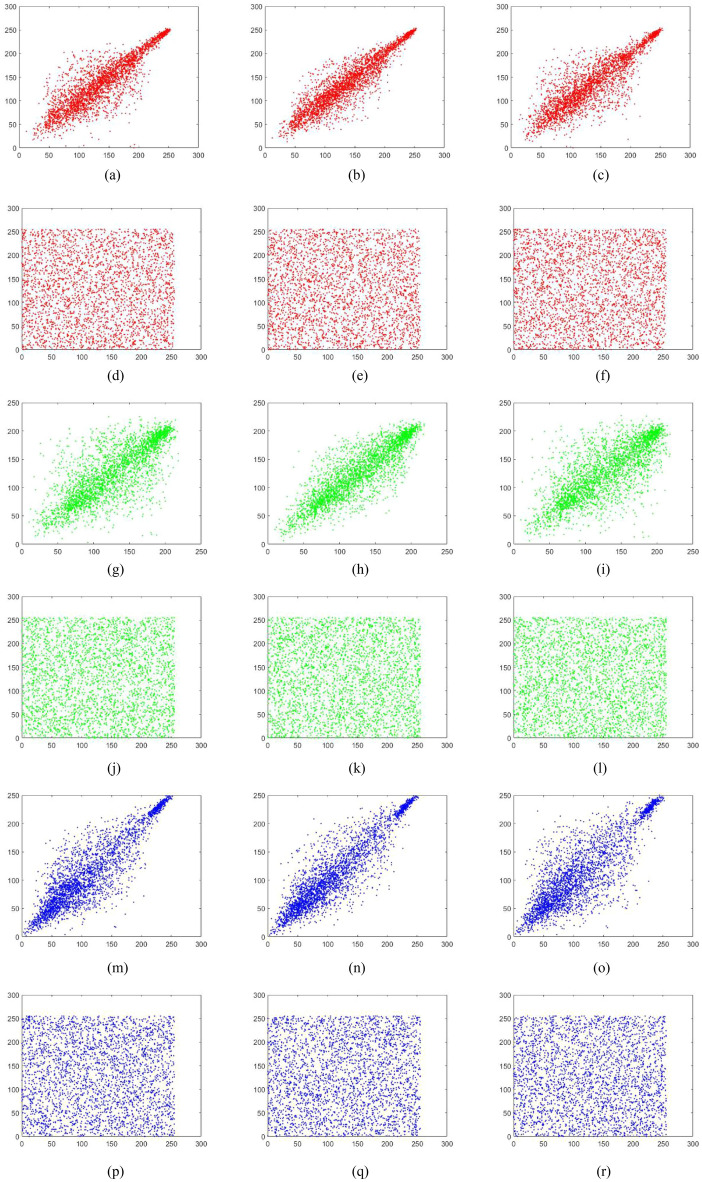
Distribution of adjacent pixels of the color image 4.2.03 and its encrypted image. (**a**) Horizontal direction of R channel of the color image 4.2.03, (**b**) vertical direction of R channel of the color image 4.2.03, (**c**) diagonal direction of R channel of the color image 4.2.03, (**d**) horizontal direction of R channel of encrypted color image 4.2.03, (**e**) vertical direction of R channel of encrypted color image 4.2.03, (**f**) diagonal direction of R channel of encrypted color image 4.2.03, (**g**) horizontal direction of G channel of the color image 4.2.03, (**h**) vertical direction of G channel of the color image 4.2.03, (**i**) diagonal direction of G channel of the color image 4.2.03, (**j**) horizontal direction of G channel of encrypted color image 4.2.03, (**k**) vertical direction of G channel of encrypted color image 4.2.03, (**l**) diagonal direction of G channel of encrypted color image 4.2.03, (**m**) horizontal direction of B channel of the color image 4.2.03, (**n**) vertical direction of B channel of the color image 4.2.03, (**o**) diagonal direction of B channel of the color image 4.2.03, (**p**) horizontal direction of B channel of encrypted color image 4.2.03, (**q**) vertical direction of B channel of encrypted color image 4.2.03, (**r**) diagonal direction of B channel of encrypted color image 4.2.03.

An efficient encryption scheme should reduce the correlation between adjacent pixels in the ciphered image significantly. In order to test the image correlation, we randomly select 3000 pairs of adjacent pixels from the image to calculate the correlation coefficients of adjacent pixels in the vertical, horizontal and diagonal directions using the following equation:

10 $$\left\{ {\begin{array}{*{20}l} {r_{xy} = \frac{{{\text{cov}} (x,y)}}{{\sqrt {D(x)} \sqrt {D(y)} }}} \hfill \\ {E(x) = \frac{1}{S}\sum\nolimits_{i = 1}^{S} {x_{i} } } \hfill \\ {D(x) = \frac{1}{S - 1}\sum\nolimits_{i = 1}^{S} {(x_{i} - E(x))^{2} } } \hfill \\ {{\text{cov}} (x,y) = \frac{1}{S}\sum\nolimits_{i = 1}^{S} {(x_{i} - E(x))(y_{i} - E(y))} } \hfill \\ \end{array} } \right.,$$

where *x* and *y* represent two adjacent pixels and *S* is the total number of adjacent pixels (*x*, *y*). *E*(*x*) is the expectation of *x* and *D*(*x*) is the variance of *x*, respectively. The pixels distribution of the plain images and the cipher images in three directions are shown in Figs. 15, 16, 17, 18, 19, 20. From figures it shows that the points in the encrypted images are randomly distributed, and the correlation of the images are greatly reduced. Meanwhile, Table 3 lists the correlation coefficients of the encrypted images which values are almost close to 0.

**Table 3:** Correlation coefficient of images.

Images	Plain image			Encrypted image		
	Horizontal	Vertical	Diagonal	Horizontal	Vertical	Diagonal
Baboon	0.755125	0.873038	0.722075	0.001272	0.003794	0.000041
Boat.512	0.968964	0.929833	0.913833	0.000844	0.000740	0.001715
Image 5.2.09	0.867678	0.899460	0.812990	0.000799	0.003387	0.000807
Image 5.3.02	0.908651	0.905706	0.857320	0.000810	0.001146	0.006518
Image 3.2.25	0.859255	0.865032	0.800579	0.004669	0.000337	0.001610
Image 7.2.01	0.944085	0.963931	0.939065	0.000987	0.001759	0.005299
White	−	−	−	0.000240	0.000932	0.002655
Black	−	−	−	0.003997	0.000895	0.001642
Average (gray images)	−	−	−	0.001702	0.001624	0.002535
Binary_boat.512	0.800731	0.756863	0.717919	0.005601	0.000672	0.002708
Color image 4.2.03 (R-component)	0.871786	0.928258	0.860717	0.008662	0.003190	0.000225
Color image 4.2.03 (G-component)	0.770344	0.864914	0.745715	0.000838	0.000717	0.001455
Color image 4.2.03 (B-component)	0.873011	0.906640	0.836914	0.000982	0.000543	0.001284

### Information entropy

Information entropy is the most important criterion to evaluate the efficiency of an image encryption algorithm. We calculate the information entropy of the cipher images and the results are listed in Table 4. The entropy of encrypted images are close to 8, which proves the proposed scheme is sufficient to withstand entropy-based attacks.

**Table 4:** Information entropy of images.

Images	Plain image	Encrypted image
Baboon	7.1457094	7.9992447
Boat.512	7.1913702	7.9993406
Image 5.2.09	6.9939942	7.9993199
Image 5.3.02	6.8303295	7.9998582
Image 3.2.25	6.7326504	7.9998453
Image 7.2.01	5.6414537	7.9998351
White	−	7.9992609
Black	−	7.9992505
Average (gray images)	−	7.9994944
Binary_boat.512	−	7.9992446
Color image 4.2.03 (R-component)	7.7066718	7.9992557
Color image 4.2.03 (G-component)	7.4744316	7.9993472
Color image 4.2.03 (B-component)	7.7522172	7.9993356

### Comparison with existing algorithms

In recent years, some researchers have combined chaotic maps with optimization methods, DNA coding, S-box and mathematical transformation to propose secure and effective image encryption schemes^1,4,25,40,41^. Some encryption algorithms have improved the efficiency of encryption by optimizing the permutation and diffusion process^16,18^. In this paper, a new efficient image encryption algorithm is proposed by combining the OF-LSTMS with the 2DCML fractional-order chaotic system. By using the OF-LSTMS, the pixel position is changed while the pixel value is changed, which realizes the synchronization of permutation and diffusion. At the same time, the 2DCML fractional-order chaotic system has better chaotic ergodicity than traditional chaotic system. Compared with some recent literature, it is clear that the proposed encryption algorithm is better in performance, as shown in Table 5.

**Table 5:** Comparison with existing algorithms.

Images	Algorithm	NPCR (%)	UACI (%)	Correlation coefficient			Information entropy
				Horizontal	Vertical	Diagonal	
Baboon	Proposed algorithm	99.59793	33.54939	0.001272	0.003794	0.000041	7.9992447
Boat.512	Proposed algorithm	99.61586	33.44338	0.000844	0.000740	0.001715	7.9993406
Average	Proposed algorithm	99.61048	33.49007	0.001702	0.001624	0.002535	7.9994944
Baboon	Ref.^25^	99.6110	33.4509	0.0016	0.0020	0.0014	7.9987
Baboon	Ref.^41^	99.6322	30.6110	− 0.0125	0.0433	0.0400	7.9970
Boat.512	Ref.^1^	99.59717	–	− 0.0034	− 0.0043	− 0.0012	7.99734
Boat.512	Ref.^18^	99.6112	33.4788	− 0.0029	0.0079	0.0081	7.9993
Average	Ref.^4^	99.610122	33.459341	0.002894	− 0.003155	− 0.002298	7.997761
Average	Ref.^16^	99.556541	33.930612	0.001564	0.001831	0.001236	7.9993
Average	Ref.^40^	99.6337	28.8432	− 0.025421	0.08270	− 0.06269	7.997701

### Robustness analysis

**Figure 21:**
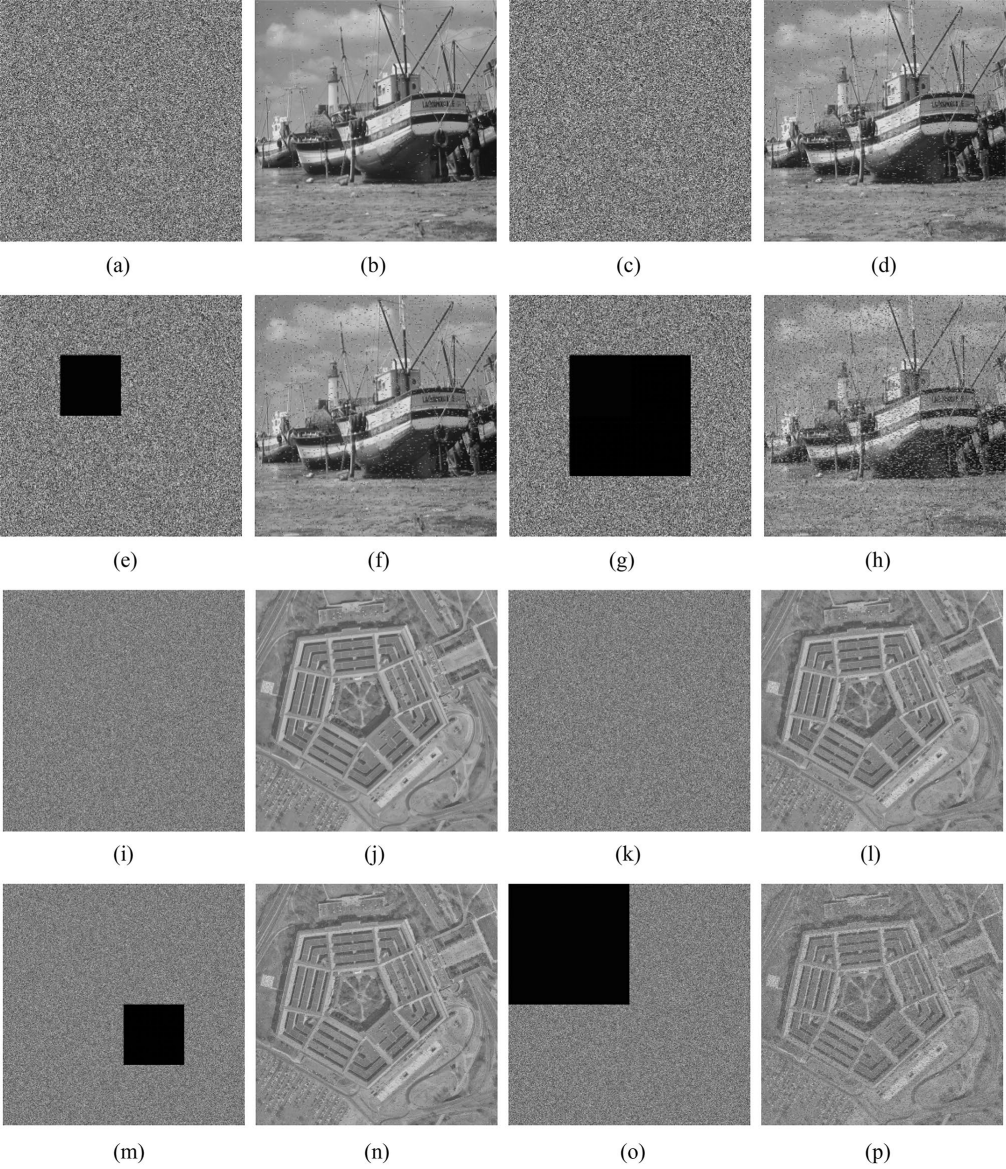
Robustness analysis results. (**a**) The encrypted image Boat.512 with salt and pepper noise, intensity 0.01, (**b**) decryption of (**a**), (**c**) the encrypted image Boat.512 with salt and pepper noise, intensity 0.05, (**d**) decryption of (**c**), (**e**) The encrypted image Boat.512 with 6% data loss, (**f**) decryption of (**e**), (**g**) the encrypted image Boat.512 with 25% data loss, (**h**) decryption of (**g**), (**i**) the encrypted image 3.2.25 with salt and pepper noise, intensity 0.01, (**j**) decryption of (**i**), (**k**) the encrypted image 3.2.25 with salt and pepper noise, intensity 0.05, (**l**) decryption of (**k**), (**m**) the encrypted image 3.2.25 with 6% data loss, (**n**) decryption of (**m**), (**o**) The encrypted image 3.2.25 with 25% data loss, (**p**) decryption of (**o**).

A feasible encryption algorithm needs the ability of anti-interference, and robustness is an important indicator^2,7,26,44^. We add Salt-and-Pepper noise with different intensities to the ciphertext images, and we also make different degrees of data loss to the ciphertext image at different locations. The decryption effects are shown in Fig. 21. The quantitative results of resisting noise and occlusion attacks are listed in Table 6. The experimental results show that the decrypted image can still display the main information of the original image, so the algorithm is anti attack.

**Table 6:** Quantitative results of resisting noise and occlusion attacks.

Images	Parameters	PSNR
Boat.512	Intensity = 0.01	30.0630
	Intensity = 0.05	22.8962
	6% data loss at the top-left corner	21.4356
	25% data loss at the center corner	15.3843
Image 3.2.25	Intensity = 0.01	30.5743
	Intensity = 0.05	22.6705
	6% data loss at the bottom-right corner	22.1602
	25% data loss at the top-left corner	16.1521

### Speed performance

The implementation environment of the proposed algorithm is Visual Studio 2019 (Visual C++) with Windows 10 Professional operating system. The specific configuration is Intel Core 2.8 GHz CPU, 8 GB RAM and 1000 GB hard disk. At the same time, MATLAB 2018 (a) is used to plot some graphs. The average running speed of encryption is 0.925 s and the encryption time of other encryption schemes^7,14,25,33^ is 2.44 s, 2.135 s, 1.067064 s and 0.9665 s, respectively. The comparison results show that our algorithm is more efficient and suitable for real-time applications.

## Conclusions

We propose a novel image encryption algorithm based on the OF-LSTMS and the 2DCML fractional-order chaotic system. The original image is divided into several image blocks which are converted into sequence data and transfer to the OF-LSTMS for encryption. Image blocks are mapped to the new position by the OF-LSTMS and chaotic sequences, which achieving the effect of the synchronous change of image pixel values and position. In addition, the 2DCML fractional-order chaotic system contains good features as larger key space, better chaotic sequences which it is more suitable for encryption algorithm. The parameters used in the input gate, the output gate and memory unit of the OF-LSTMS are initialized according to the proposed chaotic system generated, which are different from the traditional LSTM initialization method. The proposed encryption algorithm greatly reduces the time consumption and improves the efficiency of image encryption. The extensive simulated experimental results such as key sensitivity, correlation, NPCR, UACI, information entropy and robustness analysis prove that proposed algorithm is high security and efficiency for image encryption applications.
